# Gut microbial response to host metabolic phenotypes

**DOI:** 10.3389/fnut.2022.1019430

**Published:** 2022-11-07

**Authors:** Jinliang Hou, Jianguo Xiang, Deliang Li, Xinhua Liu, Wangcheng Pan

**Affiliations:** ^1^College of Animal Science and Technology, Hunan Agricultural University, Changsha, China; ^2^Changde Dabeinong Feed Co. Ltd., Changde, China

**Keywords:** metabolic phenotype, gut microbiome, nutrient metabolism, disease control, microbial metabolites

## Abstract

A large number of studies have proved that biological metabolic phenotypes exist objectively and are gradually recognized by humans. Gut microbes affect the host’s metabolic phenotype. They directly or indirectly participate in host metabolism, physiology and immunity through changes in population structure, metabolite differences, signal transduction and gene expression. Obtaining comprehensive information and specific identification factors associated with gut microbiota and host metabolic phenotypes has become the focus of research in the field of gut microbes, and it has become possible to find new and effective ways to prevent or treat host metabolic diseases. In the future, precise treatment of gut microbes will become one of the new therapeutic strategies. This article reviews the content of gut microbes and carbohydrate, amino acid, lipid and nucleic acid metabolic phenotypes, including metabolic intermediates, mechanisms of action, latest research findings and treatment strategies, which will help to understand the relationship between gut microbes and host metabolic phenotypes and the current research status.

## Introduction

Biological metabolic phenotype is based on the analysis of cell types, biological fluids and biological tissues, and uses a variety of parameters to approximately describe the organism in a specific physiological state (1). Johnson et al. (2) included genetic, environmental and gut microbial information into the biological metabolic phenotype for the first time in 2012. This new concept can describe the biological metabolic phenotype more accurately. The so-called biological metabolic phenotype is mainly determined by the organism’s genome, intestinal flora, its environment (including stress, diet, and lifestyle) and its intake of foreign substances (including drugs, cosmetics, environmental pollution, and food) (2). It is commonly described by four indexes, including the presence or absence of metabolites, the concentration of metabolites, the ratio between metabolites, and the overall information of metabolites (1).

Gut microbes are one of the important factors that determine the biological metabolic phenotype. Their metabolites and components directly affect the host’s nutrient absorption and development, and affect the host’s health by promoting the development of the host’s epithelial tissue and the immune system (3). In turn, the host’s living environment, nutrient levels, developmental stage and health status influence the composition of the gut microbiome.

The types, concentrations, ratios and overall information of metabolites vary with the composition of gut microbes. Different microbial metabolites play different metabolic roles, and thus make the host present different metabolic phenotypes. For example, *Faecalibacterium prausnitzii* and *Eubacterium rectale* produce butyrate; Probiotics such as *Lactobacillus* and *Bifidobacterium*, and 7α-dehydroxybacteria such as *Clostridium*, exhibit bile acid resistance associated with glycolytic activation; Gram-positive bacteria are more sensitive to bile acids than Gram-negative bacteria; Bile acids directly and rapidly affect the metabolism of bacteria, including membrane damage, disruption of amino acid, nucleotide and carbohydrate metabolism, and short-term exposure to bile acids significantly affects host metabolism by altering bacterial community structure (4). Some studies have changed the relative abundance of *Escherichia*, *Romboutsia*, *Intestinibacter*, and *Clostridium* in the gut through the intervention of external drugs (metformin), which leads the alterations of the concentrations of metabolites such as carbohydrates, amino acids, and fatty acids in the gut (5). The metformin also affects energy metabolism, gluconeogenesis, and branched-chain amino acid metabolism, hindering host hypoglycemic-related metabolic pathways (5). Therefore, to study the impact of gut microbes on host metabolic phenotypes, high attention should be paid to comprehensive information on gut microbiota composition and its specific metabolites.

The gut microbiota regulates host metabolism, including carbohydrate, amino acid, lipid and nucleic acid metabolism. So far, there have been a lot of researches on the influence of gut microbes on host metabolism, but most of them focus on the dominant flora and its metabolites. Therefore, comprehensive information about the metabolic phenotype of gut microbiota to the host yet to be fully explored.

## Gut microbes influence host metabolic phenotype through metabolites

The metabolites of gut microbes are very rich, such as a variety of short-chain fatty acids (acetate, propionate, and butyrate, etc.), alcohol, carbon dioxide, and hydrogen. They can utilize their respective metabolites to ensure gut viability and influence the development of the host immune system, homeostasis, and function through nutrient- and metabolite-dependent mechanisms (Table 1) (6). For example, *Blautia* spp. can convert carbon dioxide plus hydrogen to acetate; *Methanobrevibacter* convert carbon dioxide plus hydrogen to methane; *Desulfovibrio* convert sulfate to hydrogen sulfide (7, 8). These metabolites directly or indirectly affect the response of host immune and promote or delay the occurrence of their own diseases.

**TABLE 1:** Gut bacteria and the metabolites they contribute.

Metabolites	Related bacteria	Potential biological functions	References
Short-chain fatty acids: propionate, butyrate, acetate, hexanoate, valerate	*Faecalibacterium prausnitzii*, *Eubacterium rectale*, *Clostridium butyricum, Blautia* spp., *Bifidobacterium*, *lactobacillus*, *Allobaculum*, *Roseburia*, *Butyrivibrio*, *Dorea*, and *Blautia genera*	Provides energy for body functions and is associated with cholesterol synthesis, inflammation, obesity, imbalances in glucose metabolism, colorectal cancer and insulin resistance.	(7, 8, 13, 16–18, 20–22, 187, 214)
Bile acids	*Lactobacillus*, *Bifidobacteria*, *Clostridium*, *Clostridioides*, *Lactobacillus rhamnosus*, *Akkermansia muciniphila*, *Bifidobacteria*, *Enterobacter*	Regulates the balance of triglyceride, cholesterol and glucose metabolism, maintains intestinal barrier function and promotes lipid metabolism and absorption.	(4, 30, 34, 180)
Phenolic, benzoyl, and phenyl derivatives: 5-hydroxytryptophan, phenylalanine, tyrosine	*Lactobacillus*, *Some species of the genera Clostridium*, *Rumenococcus and Eubacterium.*	Related to tryptophan metabolism, brain-gut axis.	(174–176, 197, 206)
Indole derivatives: indoleacetate, indole, melatonin, serotonin, 5-hydroxyindole	*Lactococcus*, *Lactobacillus*, *Streptococcus*, *Escherichia coli, and Klebsiella*	Implicated in gastrointestinal pathologies, brain-gut axis, and a few neurological conditions.	(137, 179, 180)
Lipids: triglycerides, cholesterol, Lipopolysaccharide	*Lactiplantibacillus*, *Lactobacillus rhamnosus*, *Akkermansia muciniphila*, *Bifidobacterium*, *Klebsiella*, *Lactobacillus*	Cholesterol is the basis for sterol and bile acid production. Lipopolysaccharide induces chronic systemic inflammation; Regulate carbohydrate and lipid metabolism.	(21, 22, 35, 38, 39, 62, 75, 76, 101)

Fiber and bacterial metabolites affect host and gut health by modulating inflammation, glucose and lipid metabolism (9). For example, bacterial metabolites short-chain fatty acids have anti-inflammatory effects: Butyrate produced by *Clostridium butyricum* (10) stimulates intestinal epithelial cells and antigen presentation to produce cytokines such as TGF-β, IL-10, IL-18, (11) and reduces mild inflammation, glucose metabolism imbalance and insulin resistance in the host (12); Propionate can fight lipogenesis, lower cholesterol (13), inhibit colon cancer cell proliferation (14, 15) and induce T cells to differentiate into T regulatory cells (16). At the same time, it can control weight by stimulating satiety (17), and be absorbed by intestinal epithelial cells to improve the integrity of host intestinal epithelial cells (18). Propionate alone also reduces intra-abdominal tissue hyperplasia and lipid content in liver cells in overweight adults (19). Studies have confirmed that gut microbes can produce propionate by metabolizing carbohydrates such as *L-rhamnose*, *D-tagatose*, *Resistant starch*, *Inulin*, *Polydextrose*, *Arabinoxylans*, *Arabinoxylan oligosaccharides*, *Mannooligosaccharides*, and *Laminarans* (17); Acetate and propionate can inhibit Toll-like receptor (TLR4) stimulation to mediate the production of proinflammatory cytokines (16, 20), and the ratio of propionate to acetate determines whether propionate inhibits the conversion of acetate to cholesterol and fat (21, 22); Pyruvate mainly comes from carbohydrate metabolism, and can be further metabolized by the microbiota into succinic acid, lactic acid or acetyl-CoA to produce short-chain fatty acids, which provide energy for the normal functioning of the body (16, 18).

When the metabolites interacting with the intestinal flora are disordered, it also leads to increased intestinal permeability, bacterial endotoxin and increased harmful substances absorbed into the liver through the portal system, thereby affecting the metabolism of carbohydrates and lipids in the liver and exacerbating the imbalance between pro-inflammatory factors and anti-inflammatory effectors (23–25). Eventually, it leads to the development of metabolic fatty liver disease, infectious diseases and certain neurological diseases (26–29). For example, The deconjugation, oxidation/epimerization, (7-α-) dehydroxylation and esterification of bile acids (30) by the intestinal microbiota can dramatically change their physicochemical properties and subsequently affect their microbial toxicity and intestinal absorption (31). In addition, the metabolites enhance intestinal reabsorption by uncoupling BAs through microbial bile salt hydrolase (32); Some probiotic bacteria such as *Lactobacillus* and *Bifidobacterium* and 7α-dehydroxylating bacteria such as *Clostridium scindens* show bile acid resistance that is associated with activation of glycolysis (4).

The use of antibiotics can disrupt the gut microbiota structure and the formation of microbial metabolites, eventually affecting the host health. For example, the antibiotics can disrupt the commensal microbiota that converts primary bile acids into secondary bile acids (33–35), and the gradual accumulation of primary bile acids promotes the germination of *Clostridioides difficile spores* (a spore-forming Gram-positive bacterium and the causative agent of antibiotic-associated diarrhea), bacterial replication and the production of colitis-mediating enterotoxins, which induce host diarrhea and enteritis (31). Importantly, the gut microbial metabolites are one of the important components of host immune system. A large number of studies have shown that the metabolic phenotype of most hosts could be observed during the changes of intestinal microorganisms and their metabolites.

## Gut microbial responses to carbohydrate metabolism phenotypes

Many gut microbes can directly metabolize carbohydrates. *Lactobacillus plantarum* presents a stronger carbohydrate utilization capability (36–39), which also plays a certain role in maintaining the balance of gastrointestinal flora, improving self-immunity of host, promoting effective absorption of nutrients, reducing cholesterol content, and alleviating lactose intolerance (40). In addition to metabolizing exogenous carbohydrates by gut microbes, endogenous carbohydrates released in the gut mucus are also a constant source of nutrients for the microbiota, which are decomposed and used as components for synthesizing bacterial cell walls, thereby affecting host mucosal immunity (41); Host intestinal epithelial cells also obtain nutrients from microbial metabolism. When nutrients are deficient, microbes undergo autophagy, and some bacteria have evolved a sugar-decomposing lifestyle to escape competition (18). This direct metabolism can not only supply the growth and development needs of the host, but also enhance the disease resistance of the host to a certain extent.

Gut microbes are one of the important sources of carbohydrate metabolizing enzymes. Carbohydrate metabolizing enzymes are highly correlated with the abundance of certain gut flora (e.g., *Bacteroides*, *Prevotella*) (42). Studies have reported that a large number of carbohydrate-activating enzymes are found in the human gut flora, such as *Bacteroides thetaiotaomicron*, which has 260 glycoside hydrolases in its genome (43, 44). Human cells rarely produce these enzymes, which rely on gut microbes for energy production from remaining complex carbohydrates (45, 46). Gut microbes are not only dependent on the host, but also influence the balance of the host gut microbial ecology. Intestinal microorganisms produce metabolic enzymes that improve the host’s ability to digest complex carbohydrates and promote the growth and reproduction of the corresponding flora.

The structure, metabolites and derived metabolites of the host gut flora are altered by the ingestion of different types and levels of carbohydrates, which in turn produce different metabolic phenotypes. The gut microbiota is very sensitive to the subtle structural differences between insoluble (47) and soluble (48) plant fibers, and different fiber structures correspond to specific microbial taxa (49). Shi et al. (50) found that supplementation with microbially available carbohydrates ameliorated cognitive impairment in obese mice induced by chronic high-fat and fiber-deficient diets. Low-fiber diets cause increased by-products of proteolytic fermentation, resulting in altered microbiota and its derived metabolites (increased fecal branched-chain amino acids and decreased short-chain fatty acids), impaired colonic epithelium and intestinal mucosal detoxification, and increased inflammation (51, 52). The long-term low-carbohydrate diet in traditional Western societies has led to changes in the composition of the intestinal flora. The production of short-chain fatty acids in the metabolites of the intestinal flora is less, which cannot meet the requirements of reducing inflammation, and the incidence of diseases is significantly increased (53). Increasing the content of resistant starch (RS) in the diet increases the content of short-chain fatty acids in the human gut and feces, and the gut microbiota also changes (54, 55). For example, adding RS2 can increase the abundance of *Eubacterium rectale* and *Ruminococcus bromii* (56, 57); RS3 can alter the abundance of *Lachnospiraceae*, *Faecalibacterium*, *Alistipes*, and *Bifidobacterium*; RS4 can increase the relative numbers of *R. bromii* L2-63, *Parabacteroides distasonis* 8503, *E. rectale* 17629, and *B. adolescentis* IVS-1 (47); RS5 has potential prebiotic activity, and addition of RS5 significantly increases the relative abundance of *Bifidobacterium*, *Dialister*, *Collinsella*, *Romboutsia*, and *Megamonas* (58). Recent evidence from molecular ecology has also shown that the amount and type of non-digestible carbohydrates (e.g., resistant starch, non-starch polysaccharides, and prebiotics) influences the species composition of the intestinal microbiota both in short-term dietary interventions and in response to habitual long-term dietary intake (59). For example, supplementation of the diet with specific polysaccharides can promote the growth of *Bifidobacteria*, *Lactobacilli*, or butyric acid-producing bacteria, short-chain fatty acids production, lowering pH, and lead to inhibitory effects toward pathogens (9); A diet with low fermentable oligosaccharides, disaccharides, monosaccharides and polyols (FODMAP) diet can enrich a large amount of sugar catabolizing bacteria, reduce the frequency of abdominal pain, and treat intestinal allergies (60); Study has also shown that gut microbiome biomarkers might be associated with low FODMAP diet efficacy (42). Different types of carbohydrates select the corresponding intestinal dominant flora for the host, producing different metabolites that affect the host’s development and immune system. Resistant starch can reduce the proportion of pathogenic bacteria to a certain extent and enhance the host’s resistance to disease, which is promising in cultivating intestinal probiotics and regulating host immunity.

The latest research reports that the link between the genes encoding carbohydrate-active enzymes and the host can be used as a tool to predict whether carbohydrates can be metabolized, and guide the restoration of the ecological balance of the host’s intestinal flora, transplantation of flora and supplementation of probiotics (61). The correlation analysis between the expressions of regulatory genes and sugar metabolism genes showed that some regulatory genes were correlated with most of the sugar metabolism genes, suggesting that some two-component systems might be involved in the regulation of sugar metabolism (62). Jia et al. (12) also found that individuals with type 2 diabetes (T2D) also had altered gut microbiota. And the detection of genes related to sugar and amino acid metabolism in their gut microbiota found that the abundance of related genes was significantly reduced, indicating that the abundance of these depleted genes can be used as potential biomarkers to identify obese individuals at high risk of developing T2D (12).

## Gut microbial responses to lipid metabolism phenotypes

Lipid metabolism includes four categories: triglyceride metabolism, phospholipid metabolism, cholesterol metabolism and blood lipid metabolism, and one of the metabolic pathways is blocked, it can cause lipid metabolism disorders (63). Disorders of lipid metabolism can cause damage to host vascular endothelial cells, abnormal proliferation of smooth muscle cells, enhanced coagulation activity, inhibition of the fibrinolytic system (64), and excessive levels of total cholesterol or triglycerides in serum (65). The most intuitive phenotypes of lipid metabolism disorders in host are obesity and hyperlipidemia (64), which induce thrombosis and complications such as atherosclerosis, coronary heart disease and other cardiovascular and cerebrovascular diseases (66).

The role of gut microbes in host lipid metabolism is irreplaceable. It regulates the absorption and metabolism of lipids in the host, mainly by affecting bile acid metabolism, producing short-chain fatty acids and regulating the enteroendocrine system. Clinical studies have found that *Lactobacillus rhamnosus* is a classic probiotic, which can improve metabolism-related fatty liver disease by regulating intestinal flora, improving intestinal mucosal barrier and lowering cholesterol (67). Oral *Akkermansia muciniphila* supplementation can significantly improve insulin sensitivity and reduce insulinemia and total plasma cholesterol (68); Abnormal levels of one or both of *Bifidobacterium* and *Bacteroidetes* in the intestinal flora can cause hyperlipidemia (69–71); With the decreasing abundance of probiotics such as *Bifidobacterium*, *Lactobacillus* and *Faecalibacterium* genus in the intestines of most patients with hyperlipidemia and the increasing content of *Enterobacteriaceae* family and *Enterococcus* genus the lipopolysaccharide in the body will accumulate due to increased secretion or slowed metabolism (66, 72). Then, a part of the accumulated lipopolysaccharide enters the blood to cause endotoxemia and inflammatory response, and the inflammatory response also aggravates the symptoms of lipid metabolism disorder, resulting in a vicious circle (66, 72); Most diabetic patients have intestinal flora imbalance, and it will affect lipid metabolism and promote the occurrence and development of diabetes (73–75); Pig gut bacteria *Prevotella* is the core microbe that dominates fat deposition. Its abundance is positively correlated with feed intake, and significantly negatively correlated with pig carcass lean meat percentage. Induced by a high-fat diet, mice colonized with *Prevotelle* developed severe adipose tissue deposition (76). Studies have shown that *Prevotelle* can induce chronic inflammation in the body by activating Toll-like receptor 4 and mammalian target protein signaling pathway of rapamycin, hindering the normal operation of the body’s physiological metabolism, thereby aggravating fat inflammation and deposition (77). Lipid metabolism is an important and complex biochemical reaction in the host. Gut microbes can play a role in regulating lipid metabolism, both directly by breaking down lipids and through the production of other metabolites.

Prebiotics or probiotics can modulate lipid metabolism to improve metabolic syndrome and treat diseases such as obesity and diabetes associated with dysbiosis of the gut microbiome (78, 79). Prebiotics can promote the growth of beneficial bacteria such as *Bifidobacteria* and *Lactobacillus*, which are metabolized into lactic acid and short-chain fatty acids in the large intestine, improving host physiology, especially gastrointestinal health (80–82); Studies by Cani et al. (83) have shown that the regulation of intestinal flora by prebiotics such as *Fructooligosaccharides* can increase the content of *Bifidobacteria* in the host intestine and prevent hyperlipidemia-induced metabolic diseases such as diabetes and obesity.

The lipid structure of the diet affects the composition of the gut microbiota, which in turn affects lipid metabolism and host health. High-fat diet has been recognized as a major determinant of obesity, and the gut microbiota plays an important role in this phenotype (84). High-fat diet can induce enrichment of opportunistic pathogens such as *Betaproteobacteria* (85), *Clostridium bolteae*, *Desulfovibrio* sp. (86), and *Enterobacter cloacae* (87); Regular consumption of functional foods rich in fiber, polyphenols and polysaccharides can reduce the risk of cardiometabolic diseases, and their metabolites may inhibit pathogenic bacteria and stimulate the growth of beneficial bacteria (88); Caesar et al. (89) validated that pro-inflammatory *Bilophila wadsworthia* thrived on a lard-based diet, while *Lactobacillus* and *Akkermansia muciniphila* thrived on a fish oil-based diet; Feeding Auricularia auricula polysaccharid can increase the abundance of intestinal flora in rats, such as Parabacteroides (90), and Auricularia auricula polysaccharid can also play a role by inhibiting the absorption of external fat and enhancing the metabolism of liver fat (91); Lesel et al. (92) fed rainbow trout with low-fat and high-fat diets and found that the main bacterial groups in the feces of the low-fat group were only *Acinetobacter* spp. and *Enterobacteria*, while the high-fat group had higher bacterial diversity, mainly including *Acinetobacter* spp., *Enterobacteria*, *Flavobacterium* spp., *Pseudomonas* spp., and *Coryneforms*; In the high-fat-fed mice, the content of *Bacteroidetes* increased significantly at 1 week, and the ratio of *Firmicutes* to *Bacteroidetes* changed from 0.86 to 1.77 after 8 weeks. At the same time, fat deposition and intestinal dysbiosis were also found, the content of *Verrucobacterium* was significantly increased, while the *E. coli* was significantly decreased (93); Recent studies have shown that fasting can reduce host serum lipid levels and improve hepatic steatosis by reducing the ratio of *Firmicutes* to *Bacteroidetes* and increasing the abundance of *Allobaculum* in the host, resulting in improving metabolic disorders and intestinal flora imbalance caused by high-fat diet (94). The effects of an imbalance in the host’s flora reflect the problems caused by an imbalance in diet, and a balanced diet is one of the ways in which the host can ensure that all of its physiological functions are functioning properly. Too high or too low a lipid intake will eventually upset the existing balance.

External environmental conditions affect the structure of gut microbiota, which in turn affects host lipid metabolism. Some studies have found that the structure of the intestinal flora of carp exposed to copper has changed significantly, and the abundance of some short-chain fatty acid-producing species (such as *Allobaculum*, *Blautia*, etc.) has decreased significantly (95). Moreover, liver fat synthesis genes were significantly down-regulated, and the expression of lipolysis-related genes was significantly increased. It can be inferred that there is a significant correlation between changes in intestinal flora structure and lipid metabolism (95); Under chronic hypoxia, the intestinal microbiota composition and mucosal morphology of *Macrobrachium nipponense* are changed, which affects the metabolic enzyme activity of liver, reduces the content of intestinal short-chain fatty acids, affects the immune enzyme activity and inhibits the immune response, thus affecting the intestinal health (96).

Artificial regulation of intestinal flora structure can effectively improve host lipid metabolism and control obesity. Serum lipid levels in patients with hyperlipidemia are significantly correlated with changes in intestinal dominant flora, suggesting that adjusting the structure of intestinal dominant flora can improve serum lipid levels in patients (66). Antibiotics have been reported to disrupt gut microbial structure and gut immune cell composition, resulting in abnormal lipid metabolism. This reflects the importance of promoting antibiotic-free farming in modern farming, which is the ultimate choice in microbial treatment strategies. *Monascus vinegar* can lower body weight, total cholesterol, and triglycerides as well as ameliorating hyperlipidemia by regulating Peroxisome proliferator-activated α (PPARα)-, Nuclear factor-E2-related factor 2 (Nrf2)-, and Nuclear factor κB (NF-κB)-mediated signals and modulating the gut microbiota composition in hyperlipidemia rats (97); *Octylphenol* is a widely distributed endocrine disruptor, which can affect the expression level of genes related to fat digestion and absorption, and change the structure of intestinal flora, gradually reduce the ratio of Firmicutes/Bacteroidetes, and destroy lipid metabolism (98). The artificial addition of biological agents to alter the microbiological structure of the gut to improve disease is increasingly recognized. The ratio of *Firmicutes* to *Bacteroidetes* in the gut microbiota of patients with type 2 diabetes is altered to absorb higher energy for insulin resistance (45, 46); Regulating the content of glucagon and insulin in blood can also improve lipid metabolism disorder and intestinal flora structure, effectively preventing and treating type 2 diabetes (99). Currently, diabetes is treated with extracorporeal insulin injections. Finding insulin-secreting microorganisms and colonizing them in the gut would be a historic advance in the fight against diabetes.

## Gut microbial responses to amino acid metabolic phenotypes

Intestinal microorganisms can extensively metabolize a variety of amino acids, such as Histidine, Lysine, Threonine, Arginine, Glutarnine, Leucine, Isoleucine, and Valine, etc., with a metabolic rate of more than 50% (100, 101). When the host metabolizes amino acids, gut microbes secrete enzymes to assist amino acid metabolism, while amino acid metabolites of some microorganisms [e.g., histamine; immunomodulatory signaling; alpha-aminobutyric acid (102); putrescine] can also be involved in immunity, anti-inflammatory and other pathological mechanisms (such as improving obesity, diabetes) (103). The metabolize of amino acids by gut microbes also contribute to the survival and value-added of the microbiota and the further synthesize bacterial components (104).

Gut microbes can synthesize a variety of amino acids. Dietary amino acids can be absorbed and metabolized by intestinal cells for life activities and protein synthesis, and can also be metabolized and decomposed by intestinal microbes to maintain the ecology of intestinal microbes, meet the host’s amino acid requirements, and regulate the host’s inflammation and immune levels. Gut microbes such as *Streptococcus bovis, Selenomonas ruminantium*, and *Prevotella bryantii* can synthesize amino acids at physiological peptide concentrations and integrate them into host proteins (105, 106); The most abundant BCAAs, valine, isoleucine and leucine, are essential amino acids synthesized by plants, fungi and bacteria, particularly by members of the gut microbiota (107); Gut bacteria can produce aspartic-, cysteine-, serine-, and metallo-proteases, in a typical fecal sample, these bacterial enzymes are far outnumbered by proteases arising from human cells (108).

The difference in the amount of essential amino acids measured by N balance method and tracer-derived statistics also indicates that in addition to diet, the gastrointestinal microbiota can also synthesize essential amino acids to meet host requirements (109). Gut microbes synthesize amino acids not only to deliver energy but also to maintain a reduced cofactor cycle (109).

Amino acids and their metabolites are integral components of host nutrition and synthetic immunity, and they play a role in maintaining mammalian homeostasis by regulating protein synthesis, glucose and lipid metabolism, insulin resistance, hepatocyte proliferation, and immunity (107). For example, L-tryptophan (Trp) is an important component of protein, and can be converted into a variety of substances (such as serotonin, melatonin, indole, kynurenine) as a metabolic substrate. It plays an important role in the onset of neurological diseases and immune regulation such as intestinal tolerance, balance between intestinal microbiota, hinder depression, chronic fatigue syndrome and physical mobility disorder (110–113); Endogenous Trp metabolites (kynurenines, serotonin, and melatonin), and bacterial Trp metabolites (indole, indolic acid, skatole, and tryptamine) also have profound effects on gut microbial composition, microbial metabolism, the host’s immune system, the host-microbiome interface, and host immune system–intestinal microbiota interactions (114); Aromatic amino acids are metabolized by gut microbes and their products can serve as signaling molecules in host physiology (115).

Try metabolism has a central role in host health. Host genes can directly or indirectly modulate the production of microbial Trp metabolites and affect the composition and function of the gut microbiota (114). Shotgun metagenomics data shows that host hepatic steatosis and metabolism are associated with an imbalance in aromatic and branched-chain amino acid metabolism (116). The phenotype of host metabolic disturbances in the gut microbiota has been found preclinically and clinically to be a reduced ability of microorganisms to metabolize tryptophan as an aromatic hydrocarbon agonist (117). When the activation of the aromatic hydrocarbon pathway is defective, host GLP-1 and IL-22 production is reduced, intestinal permeability is altered, and lipopolysaccharide translocation is promoted, leading to host inflammation, insulin resistance, and hepatic steatosis (118). When changes in host tissue homeostasis require an immune response, gut microbiota can directly or indirectly activate host aryl hydrocarbon receptors and pregnane X receptors through derivatives produced by tryptophan metabolism (indole and its compounds) (119). A tryptophan-rich diet increases Aryl Hydrocarbon Receptor (AhP) mRNA expression and activates AhP (114). AhR can mediate exogenous metabolism, rely on IL-22 activation in the gut, and influence the balance between immunity and microbiota by modulating microbial composition through antimicrobial peptides (120–122). On a low-tryptophan diet, tryptophan metabolites were reduced, and mice or piglets were prone to inflammation (123). After adequate supplementation, metabolites were increased, inflammation was significantly reduced, and the degree of dextran sodium sulfate-induced colitis was reduced (121, 124, 125).

In turn, the composition of microbiota can influence the production of Trp microbiota-derived metabolites, affect the host’s intestinal homeostasis and lead to the loss of intestinal homeostasis and intestinal inflammation (114). Specific gut bacteria determines the availability of Trp to the host and then regulates serotonin and subsequent melatonin synthesis (126). Serotonin is a monoamine neurotransmitter involved in the regulation of central nervous system transmission and intestinal physiological functions, while tryptophan is the only precursor for serotonin production. Melatonin, a powerful anti-inflammatory factor, is also produced through tryptophan metabolism and can alleviate increase gut permeability and immune activation caused by *Escherichia coli* (127–130).

Various preclinical experimental strategies emphasize that both tryptophan availability and 5-HT signaling are profoundly affected by gut microbiota composition (131), and intervention targeting the microbiota can modulate the tryptophan metabolites kynurenine, tryptamine, indole, serotonin, and melatonin, which has important implications for the treatment of brain-gut axis diseases (127, 129). Gut microbiota derivatives reduce the ratio of kynurenine to tryptophan in blood in the regulation of tryptophan metabolism, suggesting that lack of gut microbiota reduces the activity of host Indoleamine 2,3-dioxygenase, which affects color kynurenine pathway of amino acid (118, 132). Gut microbiota can also influence tryptophan degradation and tryptophan cycling in the host through kynurenine pathway (131), and mitigate the adverse effects of aging on the central nervous system (133–136).

The dietary structure of amino acids affects the host gut microbial structure, which in turn affects host metabolism and health. Studies have shown that the foul odor of pig excrement is produced by intestinal microorganisms metabolizing amino acids (137). The level of amino acids in a balanced diet can change the abundance of pig intestinal microbes, thereby improving fermentation and reducing odor-causing compounds in pig excrement (137). An increase in branched-chain amino acids induces changes in the abundance of certain gut flora, such as increased abundance of *Akkermansia* and *Bifidobacterium* in mice supplemented with a mixture of branched-chain amino acids (138). The accumulation of branched-chain amino acids is positively correlated with insulin resistance. The number of bacteria that absorb branched-chain amino acids (such as *Butyrivibrio crossotus* and *Eubacterium siraeum*) gradually decreases in insulin-resistant patients, resulting in the accumulation of branched-chain amino acids and an insulin-resistant phenotype (139). Metabolism of proline is associated with disturbances in host emotional expression. Proline is the dietary factor that has the greatest impact on depression, and individual depression levels are enhanced with increased proline intake and plasma proline levels, which are not significantly associated with antidepressants (140). The higher the proline content in plasma, the lower the proline recycling-involved in glutamate, gamma-aminobutyric acid receptor activation, synaptic interactions, and axon guidance pathways. Likewise, the higher abundance of *Parabacteroides* and *Acidaminococcus* species and lower abundance of Bifidobacterium and short-chain fatty acid-producing species (such as *Roseburia*, *Butyrivibrio*, *Dorea*, and *Blautia genera*) in the gut (140). If mice are fed with a diet deficient in tryptophan, the gut microbiota will be dysregulated and gut immunity will be compromised (130).

Gut microbes can affect host health by affecting protease activity. The protease activity in the intestine is not only affected by the environment such as temperature and pH, but also restricted by microorganisms such as *Bacteroides*, *Clostridium*, *Propionibacterium*, *Fusobacterium*, *Streptococcus*, and *Lactobacillus* (141). Affected by various factors such as heredity, diet, medication, the host has respective characteristic of intestinal flora. Under normal circumstances, intestinal flora and host can benefit from this kind of dynamic balance. Once this balance is broken, the body of the peroxidase proliferation of activated receptors (PPARs) signaling pathway and amp activated protein kinase (AMPK) path are blocked (142, 143). The lack of proteases in the host will lead to increased susceptibility to disease, such as lack of angiotensin I-converting enzyme 2 (Ace2), which will lead to increased susceptibility to intestinal inflammation caused by intestinal epithelial cells (123).

## Gut microbial responses to nucleic acid metabolic phenotypes

Nucleic acids in food mostly exist in the form of nucleoproteins, which are broken down into nucleic acids and proteins in the stomach. Nucleotide is the basic unit of nucleic acid. Its main functions are the raw material for nucleic acid synthesis, the utilization form of energy in the body, the participation in metabolism and physiological regulation, the component of coenzyme, the intermediate product of activation, etc. The assembly of nucleotides has a high metabolic demand and requires substances such as glucose, aspartic acid, serine, glycine, CO_2_, and glutamine for synthesis. These substances supplement the carbon and nitrogen sources for nucleotide synthesis through major pathways such as glycolysis, the serine-glycine pathway, the glutamine aminotransferase reaction, the Krebs cycle without or with anaplerotic inputs, and the pentose phosphate pathway (144).

Nucleic acid substances are considered to be an very important class of molecules in organisms because they have function of heredity, mediating and catalyzing biochemical reactions, providing or transferring energy, etc. (145). Lack of nucleotides in food can damage the host liver (146, 147), gut (148–151) and immune system (152, 153). Exogenous addition can promote the maturation, activation and proliferation of lymphocytes, improve the phagocytosis of macrophages (154, 155), promote the balance of intestinal flora (such as increasing the proportion of *Bifidobacteria*), and reduce the proportion of pathogenic bacteria (145). Exogenous nucleotides have an important impact on maintaining the normal function of the host immune system, intestinal function, improving the structure of the intestinal flora and lipid metabolism, and will have a broad prospect and development space in the field of medical and health care.

The intestinal flora is one of the important “organs” for the host to absorb and utilize nucleic acids. For example, the nucleosidase produced by *Ochrobactrum anthropi* in the human intestine can decompose purine nucleosides in food, and also promote other enzymatic reactions (156–158). *Escherichia coli* and baker’s yeast (dietary nucleotides) can convert glucose to 2-deoxyribose 5-phosphate and generate the desired substrate for 2-deoxyribose 5-phosphate, respectively (159).

Nucleotides and nucleosides strongly dose-dependently modulate infant gut microbiota composition and metabolic activity. Nucleotide and nucleoside are added in the infant formula, increasing the amount of the anaerobic bacteria, gastrointestinal bacteria, *Fusobacterium*, *Lactobacillus*, *Staphylococcus*, *Leuconostoc*, increased levels of bacteria and bacteria related to nucleotide and metabolism of sulfur and iron transcription content will increase, and decreasing the biosynthesis of *Salmonella*, total factor and vitamin (160). Yeast extract (dietary nucleotides) can increase the number of *Lactobacilli* and *Bifidobacteria*, thereby increasing butyrate production to stabilize and promote the gut microbiota in the elderly (161). Numerous studies have also demonstrated that the addition of exogenous nucleotides can increase the proportion of beneficial bacteria in the host gut microflora and play a positive regulatory role.

## Signaling roles of gut microbes in host metabolism

In recent years, the gut microbiota has attracted much attention as a key mediator of brain-gut axis signaling. Brain-gut interactions are mainly divided into neural, endocrine and immune pathways. There is growing evidence that the influence of the gut microbiota extends beyond the gut, which modulates brain function and subsequent behavior through the brain-gut axis (162–164). Dysregulation of the microbiota-gut-brain axis leads to neuroinflammation and synaptic damage that contribute to obesity-related cognitive decline (50). Preclinical and clinical studies have also shown that the gut microbiota influences central nervous system physiology, anxiety, depression, social behavior, cognitive function and visceral pain (134). For example, high-fat diet-induced changes in gut microbiota can lead to cognitive impairment in mice; Mice transplanted with microbiota from obese humans also showed reduced memory scores. Moreover, RNA sequencing of the medial prefrontal cortex showed that short-term memory was associated with aromatic amino acid pathways, inflammatory genes, and bacterial communities (165). Among them, *Clostridium*, *Ruminococcus* and *Eubacterium* genera in *Firmicutes* phylum, as well as *Selenomonas* family are positively correlated with memory score, while *Bacteroidetes* phylum and *Proteobacteria* phylum species are negatively correlated with memory score (165). This suggests that gut microbes can talk to the brain through neural interactions and influence host cognition and behavior.

Gut microbiota imbalance affects hippocampal brain and nerve development by activating microglia (32). Brain-gut interactions play a role in functional GI disorders [e.g., irritable bowel syndrome (IBS)] (166), organic GI disorders to a lesser extent [e.g., inflammatory bowel disease (IBD)], and other disorders that may be associated with dysregulation of brain-gut communication (e.g., obesity and anorexia nervosa), but the specific mechanisms of the interaction are not well understood (167).

In the study of the host brain-gut axis, it was found that tryptophan and 5-hydroxytryptophan metabolic systems are involved in all levels of the brain-gut-microbiome axis. For example, germ-free mice show different social novelty preferences than normal mice (132, 168, 169); Bacterial colonization of germ-free mice after weaning normalizes this phenomenon (132, 169, 170); More importantly, bacterial colonization of weaned germ-free mice normalizes circulating tryptophan availability and kynurenine pathway metabolism (132, 171). *Lactococcus*, *Lactobacillus*, *Streptococcus*, *Escherichia coli*, and *Klebsiella* can directly use tryptophan to synthesize serotonin (127, 131). Serotonin is widely present in the gut, which can cross the blood-brain barrier and act directly on the central nervous system. At present, tryptophan treatment strategies have focused on direct regulation of the 5-hydroxytryptophan (5-HT) metabolic system, but due to the heterogeneous of the disease and the diversification of 5-HT effects, these strategies are only partially effective in distinct patient subsets with stress-related brain-gut axis disorders (131).

The gut-liver axis is another pathway by which the host controls and shapes the gut microbe to protect the gut barrier. The gut microbiome, liver inflammation and metabolism are connected by the gut-liver axis, and the important mediators between the two are the gut microbiota and microbiota metabolites (172). Gut-derived compounds, such as short-chain fatty acids, bile acids, methylamine, amino acid derivatives, and related microbial molecular patterns stimulate host energy metabolism, food intake, and regulate signaling and metabolic pathways in key tissues by sensing signals, enhancing host–gut microbiota interactions (173). Another example is that microbial tryptophan metabolites are directly involved in hepatic immune responses in liver disease, or indirectly affect function by modulating gut barrier function and signaling along the gut-liver axis (117, 174). The link between the gut and liver directly influences the composition of the gut flora and the integrity of the barrier, and is an important factor in liver function. An imbalance on either side will lead to a decrease in immunity and even disease.

In the brain-gut axis and gut-liver axis, the gut microbes directly participate in or indirectly regulate the metabolic functions of key tissues such as the brain and liver through signal transduction, thereby affecting the health of the host. At present, although the key mediators and specific mechanisms of brain-gut axis and gut-liver axis signaling are still poorly understood, it is of great significance to pay close attention to the signaling of gut microbes.

## Prospects for the study of gut microbes and host metabolic phenotypes

Various chemicals produced or modified by the gut microbiota trigger responses in various physiological functions of the host, including immune, neurological, and metabolic (175). A large number of studies have proved that the occurrence of host diseases is closely related to the diversity of intestinal flora, the difference of main representative flora, and the abundance and ratio of different flora, mainly affecting host metabolism and metabolites (144). The role and status of intestinal flora and its metabolites in monitoring host health and disease prevention and control is becoming more and more important. Furthermore, the monitoring of key indicators and the mining of therapeutic factors will be another great progress in the history of human disease prevention and control.

### Gut microbes and their metabolites may be important markers for disease screening

Numerous studies have demonstrated that changes in the abundance of gut microbiota are a common marker of neoplastic diseases, such as *Fusobacterium nucleatum*, *Escherichia coli*, *Bacteroides fragilis*, and *Aspergillus* are associated with carcinogenesis (176, 177); By comparing the gut flora of gestational diabetes patients and healthy people, it was found that gestational diabetes patients had abundant OTUs of *Lachnospiraceae* family, and decreased OTUs of *Enterobacteriaceae* and *Ruminococcaceae* families (178); Obese patients are often complicated by diabetes, and the abundances of *Ruminococcus gnavus*, different *Bacteroidetes* and *Enterobacter* species in the gut tend to increase compared with insulin resistance and obesity (179, 180); Compared with healthy people, patients with primary sclerosing cholangitis had significantly reduced gut microbiota but significantly enriched *Clostridium* species with reduced *Eubacterium* spp. and *Ruminococcus obeum* (181); And then a further study of its gut microbial genes showed that the genes related to the synthesis of vitamin B6 and branched-chain amino acid cores in gut microbes were significantly reduced (181).

Different microbial metabolites in blood can reflect different healthy aging (age) states with unique gut microbiota. In the study of the intestinal flora of physiologically aging people, the core flora of the gut microbiota of the elderly is attenuated, and the results at the genus level show that Bacteroides are mainly reduced (182). Studies have also confirmed that there are key families of microorganisms in the gut microbiota with potential therapeutic effects (183–185).

The structure of the gut microbiota and its metabolites are also markers of many diseases. Elevated ratio of Firmicutes to Bacteroidetes is regarded as a microbial marker of obesity (186); The profiles of gut microbiota and serum metabolites are potential diagnostic markers for lung cancer. Compared with healthy individuals, the feces of patients with lung cancer showed significant differences in *Megasphaera*, *Clostridioides*, *Erysipelotrichaceae*, and *Phascolarctobacterium*, which the diversity of intestinal flora in patients with lung cancer was higher. Moreover, the serum levels of certain glycerophospholipids (e.g., LysoPE, LysoPC, LysoPC), AcylGlcADG, AcylGlcADG, Acylcarnitine and hypoxanthine can be used to distinguish lung cancer patients from normal individuals (187); Microbial metabolic imbalance of aromatic and branched-chain amino acids is a metabolic hallmark that accompanies liver inflammation (188). Aromatic amino acid metabolites can be used as signal molecules in host physiology (116). For example, phenylalanine (189) metabolism can be used as a stage-specific marker for colorectal cancer, because it can be seen that the genes that metabolize Phe in the intestinal flora of patients with colorectal cancer are increased, and it is significantly increased in the gut (116); Tyrosine and tryptophan metabolism and valine, leucine, and isoleucine degradation are significantly associated with identified gut microbiota markers and osteoporosis. Ling et al. (190) found that the relative abundances of *Actinobacillus*, *Blautia*, *Oscillospira*, *Bacteroides*, and *Phascolarctobacterium* were positively correlated with osteoporosis, while some of genera of *Veillonellaceae*, *Ruminococcaceae*, and *Collinsella* were negatively associated with the presence of osteoporosis. However, further confirmation in animal or *in vitro* experiments is required as a diagnostic or therapeutic marker (190).

Inflammatory cytokines are key factors for the expression of indoleamine 2, 3-dioxygenase (IDO1) and tryptophan 2, 3-dioxygenase (TDO) (131). Moreover, it has been proposed as markers of gastrointestinal diseases in the study of mucosal inflammation and colon cancer (131).

Different types of carbohydrate-active enzymes may provide markers for the screening of certain diseases. For example, glycosyltransferases can catalyze the synthesis of glycoconjugates, including the inflammatory factor lipopolysaccharide-a common feature of many diseases. Comparing differences in carbohydrate-active enzymes in health and disease suggests that microbial carbohydrate metabolism can be a good basis for optimizing pre-selected, pro- and synergistic biological candidates (61). Most disease changes are associated with changes in gut microbial abundance, while further research is needed to prove whether differential bacteria can be used as indicators. The clarification of indicator species will provide new ideas for therapeutic strategies.

### Improving gut microbes will become an effective means of disease prevention and treatment

Gut microbes are an important factor in regulating various physiological functions, immune system, cardiovascular system, intestinal function, digestion and absorption of nutrients and their metabolites (191). Once the healthy balance of the host’s intestinal flora is disrupted, the host will become ill. On the contrary, adjusting the balance of the host’s intestinal flora will help the host maintain a healthy state. Direct supplementation of beneficial microorganisms modulates host hepatic and systemic lipid metabolism (192), energy stabilization (193), and glycemic control (194). Meanwhile, it reduces the risk of diet-induced obesity, insulin resistance, and type 2 diabetes (195).

Transplanting fecal microbiota can effectively treat some recurrent *Clostridium difficile* infections in small trial in metabolic syndrome and obesity (196); Gavage of mice with *Bacteroides thetaiotaomicron* reduced plasma glutamate concentrations and attenuated diet-induced weight gain and obesity in mice, suggesting the possibility of targeting the gut microbiota to intervene in obesity (197); In mouse experiments, it was found that *Prevotella copri* and *Bacteroides vulgatus* are the key species driving branched chain amino acids (BCAAs) biosynthesis and insulin resistance (139). In addition, using *Prevotella copri* to interfere with the intestinal flora of mice can induce insulin resistance, aggravate glucose intolerance and increase BCAAs circulation level (139). Kato et al. (198) found that oral administration of *Porphyromonas gingivalis* could alter the composition of the host gut microbiota, thereby impeding or altering the metabolic function of host amino acids, altering immune regulation and gut barrier function, and mediating systemic diseases.

Nutritional stress caused by an unbalanced diet can lead to changes in the host gut microbiome, which is one of the important factors in the occurrence of metabolic diseases such as hypertension, abnormal lipid metabolism, and vascular dysfunction (191). This makes dietary guidelines for the population, based on regional differences, particularly important. Dietary and lifestyle interventions to supplement carbohydrates needed by microorganisms can enhance the ability of intestinal microbiota to metabolize carbohydrates, reduce body weight (199), and reduce metabolic diseases caused by intestinal microbiota metabolism in overweight and obese patients. An unbalanced diet not only adds to the metabolic stress of the host’s metabolic organs, but also disrupts the balance of the intestinal microbial ecology.

More and more data suggest that host health is associated with dietary microbial and carbohydrate-induced changes in microbiota composition and diversity (53). Breast milk contains a variety of complex oligosaccharides that are not easily digested but are essential for the composition of the neonatal gut microbiota (200). The microbiota that colonize an infant’s gut are directly related to its ability to grow and develop normally in later life and to the strength of its immune system. Compared with formula, breastfed infants had higher levels of *Bifidobacteria* and lactic acid bacteria as well as lower levels of potential pathogens. By contrast, formula-fed infants had a more diverse gut flora, mainly *Staphylococcus*, *Bacteroides*, *Clostridium*, *Enterococcus*, *Enterobacter*, and *Atopobium* (201). During weaning, the phyla *Proteobacteria* and *Actinobacteria* are replaced by *Firmicutes* and *Bacteroidetes* as the dominant members of the infant microbiome due to the complementary introduction of a variety of novel food substances and nutrients (202, 203). Tanes et al. (204) found that unbalanced dietary nutrition can lead to an imbalance of gut microbiota, and dietary fiber plays an important role in the restoration of gut microbiota, but the mechanism remains unclear. Flint found that both the amount and type of indigestible carbohydrates (such as resistant starch, non-starch polysaccharides, and prebiotics) affected the species composition of the gut microbiota during both short-term dietary interventions and long-term dietary habits (59). Different geographic regions have different dietary habits, and there are also significant differences in the composition of gut microbes. Compared with Europeans, Africans consume approximately twice as much dietary fiber, have a higher diversity of gut microbes, produce more short-chain fatty acids, and are more abundant in *Bacteroidetes* relative to *Firmicutes* (179, 205).

It is clear that differences in food culture also reflect the consistency of nutritional needs from the dietary guidelines of different countries^1^. All guidelines recommend limiting or avoiding foods with high added sugar, salt, and saturated fat (206). Some countries specifically mention avoiding processed, ultra-processed and/or packaged foods (206).

## Conclusion

The metabolic phenotype of organisms exists objectively (207), and is gradually being recognized and deciphered by humans. Gut microbes are the second digestive organ in the host and one of the key factors determining the host’s metabolic phenotype (2). They directly or indirectly participate in the host’s metabolism, physiology, and immunity through population structure changes, metabolite differences, signal transduction, and gene expression, helping the host to resist disease and maintain health. A large number of studies have confirmed that finding new and effective ways to prevent or treat host metabolic diseases through the association of gut microbiota with host metabolic phenotypes is not only feasible and will be a historic step in the study of gut microbiota.

But gut microbiota composition and function are also influenced by host genetics (208), diet (209), age (210, 211), mode of birth (212), geography (210), host immune status (213), travel (214), and drug use (215, 216). How to obtain comprehensive information on the impact of gut microbes on host metabolic phenotypes? What are the mechanisms underlying the role of gut microbes in host metabolic phenotypes? Can we find the key regulators and indicators that gut microbes regulate host metabolism? These issues would be the focuses of future research on gut microbes.

The gut microbiota and metabolites are not only the monitors of host disease, but also the guardians of maintaining and stabilizing host health. Although the existing researches still focus on the simple flora structure and the level of a few influencing factors, the theoretical concept that gut microbes affects the host’s metabolic phenotype provides a good research idea for the systematic study of metabolic diseases caused by gut microbes. As more and more new discoveries about the metabolic phenotypes of gut microbes on the host are published, it is about to become possible to use gut microbes to maintain the metabolic health of life.

## Author contributions

JH and JX conceived the manuscript. JH wrote the manuscript. JX, DL, XL, and WP edited the manuscript. All authors read and approved the final version of the manuscript.

## Footnotes

1
http://www.fao.org/nutrition/education/food-based-dietary-guidelines
